# Nitro-Oleic Acid (NO_2_-OA) Improves Systolic Function in Dilated Cardiomyopathy by Attenuating Myocardial Fibrosis

**DOI:** 10.3390/ijms22169052

**Published:** 2021-08-22

**Authors:** Simon Braumann, Wibke Schumacher, Nam Gyu Im, Felix Sebastian Nettersheim, Dennis Mehrkens, Senai Bokredenghel, Alexander Hof, Richard Julius Nies, Christoph Adler, Holger Winkels, Ralph Knöll, Bruce A. Freeman, Volker Rudolph, Anna Klinke, Matti Adam, Stephan Baldus, Martin Mollenhauer, Simon Geißen

**Affiliations:** 1 Department of Cardiology, Faculty of Medicine and University Hospital Cologne, University of Cologne, 50937 Cologne, Germany; Felix.Nettersheim@uk-koeln.de (F.S.N.); dennis.mehrkens@uk-koeln.de (D.M.); senai.bokredenghel@uk-koeln.de (S.B.); Alexander.Hof@uk-koeln.de (A.H.); richard.nies@uk-koeln.de (R.J.N.); christoph.adler@uk-koeln.de (C.A.); Holger.Winkels@uk-koeln.de (H.W.); Matti.Adam@uk-koeln.de (M.A.); Stephan.Baldus@uk-koeln.de (S.B.); Martin.Mollenhauer@uk-koeln.de (M.M.); Simon.Geissen@uk-koeln.de (S.G.); 2 Center for Molecular Medicine Cologne (CMMC), Faculty of Medicine and Faculty of Mathematics and Natural Sciences, University of Cologne, 50937 Cologne, Germany; Wibke.Schumacher@uk-koeln.de (W.S.); nam.im1@uk-koeln.de (N.G.I.); 3 Cologne Cardiovascular Research Center (CCRC), Faculty of Medicine, University of Cologne, 50937 Cologne, Germany; vrudolph@hdz-nrw.de; 4 Department of Medicine, Integrated Cardio Metabolic Centre (ICMC), Heart and Vascular Theme, Karolinska Institute, 17177 Stockholm, Sweden; Ralph.Knoell@AstraZeneca.com; 5 Bioscience, Cardiovascular, Renal & Metabolism, BioPharmaceuticals R&D, AstraZeneca, 43150 Mölndal, Sweden; 6 Department of Pharmacology and Chemical Biology, University of Pittsburgh, Pittsburgh, PA 15260, USA; freerad@pitt.edu; 7 Agnes Wittenborg Institute for Translational Cardiovascular Research, Clinic for General and Interventional Cardiology/Angiology, Herz- und Diabeteszentrum NRW, University Hospital of the Ruhr-Universität Bochum, 32545 Bad Oeynhausen, Germany; aklinke@hdz-nrw.de

**Keywords:** nitro-oleic acid, dilated cardiomyopathy, muscle LIM protein, myocardial fibrosis, TGFβ, alpha smooth muscle actin

## Abstract

Nitro-oleic acid (NO_2_-OA), a nitric oxide (NO)- and nitrite (NO_2_^−^)-derived electrophilic fatty acid metabolite, displays anti-inflammatory and anti-fibrotic signaling actions and therapeutic benefit in murine models of ischemia-reperfusion, atrial fibrillation, and pulmonary hypertension. Muscle LIM protein-deficient mice (*Mlp^−/−^*) develop dilated cardiomyopathy (DCM), characterized by impaired left ventricular function and increased ventricular fibrosis at the age of 8 weeks. This study investigated the effects of NO_2_-OA on cardiac function in *Mlp^−/−^* mice both in vivo and in vitro. *Mlp^−/−^* mice were treated with NO_2_-OA or vehicle for 4 weeks via subcutaneous osmotic minipumps. Wildtype (WT) littermates treated with vehicle served as controls. *Mlp^−/−^* mice exhibited enhanced TGFβ signalling, fibrosis and severely reduced left ventricular systolic function. NO_2_-OA treatment attenuated interstitial myocardial fibrosis and substantially improved left ventricular systolic function in *Mlp^−/−^* mice. In vitro studies of TGFβ-stimulated primary cardiac fibroblasts further revealed that the anti-fibrotic effects of NO_2_-OA rely on its capability to attenuate fibroblast to myofibroblast transdifferentiation by inhibiting phosphorylation of TGFβ downstream targets. In conclusion, we demonstrate a substantial therapeutic benefit of NO_2_-OA in a murine model of DCM, mediated by interfering with endogenously activated TGFβ signaling.

## 1. Introduction

Dilated cardiomyopathy (DCM) is a major health issue in Western countries with high incidence and mortality rate and the most frequent cause for heart transplantation [1,2]. Ischemic heart disease is the most common cause for DCM, while up to 40% of non-ischemic DCM are caused by genetic mutations [3]. Pathognomonic features of DCM are progressive dilation and systolic dysfunction of one or both ventricles, increased myocyte apoptosis and myocardial fibrosis [4,5,6]. Transforming growth factor-β (TGFβ) is a primary stimulus of fibrosis [7], via both canonical and non-canonical signaling pathways. TGFβ induces the activation and proliferation of fibroblasts, stimulating their transdifferentiation to myofibroblasts and the secretion of profibrotic cytokines, thus leading to an excessive production of extracellular matrix proteins such as collagen I and III [8,9,10]. While anti-fibrotic drug strategies sometimes blunt the progression of idiopathic pulmonary fibrosis and angiotensin-converting enzyme inhibitors have been shown to attenuate cardiac fibrosis, no specific anti-fibrotic agent is approved for DCM treatment [11,12,13]. One emerging group of anti-fibrotic therapeutics is nitrated fatty acids (NO_2_-FA) [14].

NO_2_-FA are signaling mediators which are endogenously synthesized by the reaction of unsaturated fatty acids with nitrogen dioxide (NO_2_), a free-radical product of reactions initiated by both NO and NO_2_^−^ [15]. An electrophilic character enables NO_2_-FA to reversibly react with target proteins at specific cysteine residues via Michael-addition, termed nitro-alkylation. Nanomolar concentrations of NO_2_-FA can be found homeostatically in human plasma and urine, with concentrations increasing upon metabolic and inflammatory stress [16,17]. Continuous subcutaneous infusion of NO_2_-FA yields steady-state plasma concentrations of both free and esterified NO_2_-FA [18,19]. Nitrated oleic acid (NO_2_-OA) is a NO_2_-FA that has proven effective in animal models of various cardiovascular diseases, such as atrial fibrillation, ischemia-reperfusion damage, and pulmonary arterial hypertension (PAH) [14,19,20,21]. Due to its favorable pharmacodynamics, 10-NO_2_-OA was developed as a drug to treat fibrotic and inflammatory diseases and is currently being tested in phase II clinical trials for therapy of chronic pulmonary and renal diseases [22].

Given that myocardial fibrosis is a hallmark feature of DCM, we sought to investigate potentially beneficial effects of NO_2_-OA on this disease phenotype. Muscle LIM protein (MLP)-deficient mice lack the gene *Csrp3/Mlp* (*Mlp^−/−^*) and represent a well-established model of DCM. MLP is located at the Z-disk of terminally differentiated striated muscle cells, where it is responsible for maintenance of cytoarchitectural organization [23]. *Mlp^−/−^* mice develop age-related DCM, including key phenotypical disease features such as left ventricular dilation, reduced left ventricular function, and myocardial fibrosis [23,24].

## 2. Results

### 2.1. NO_2_-OA Improves Left Ventricular Systolic Function in Mlp^−/−^ Mice

**Figure 1:**
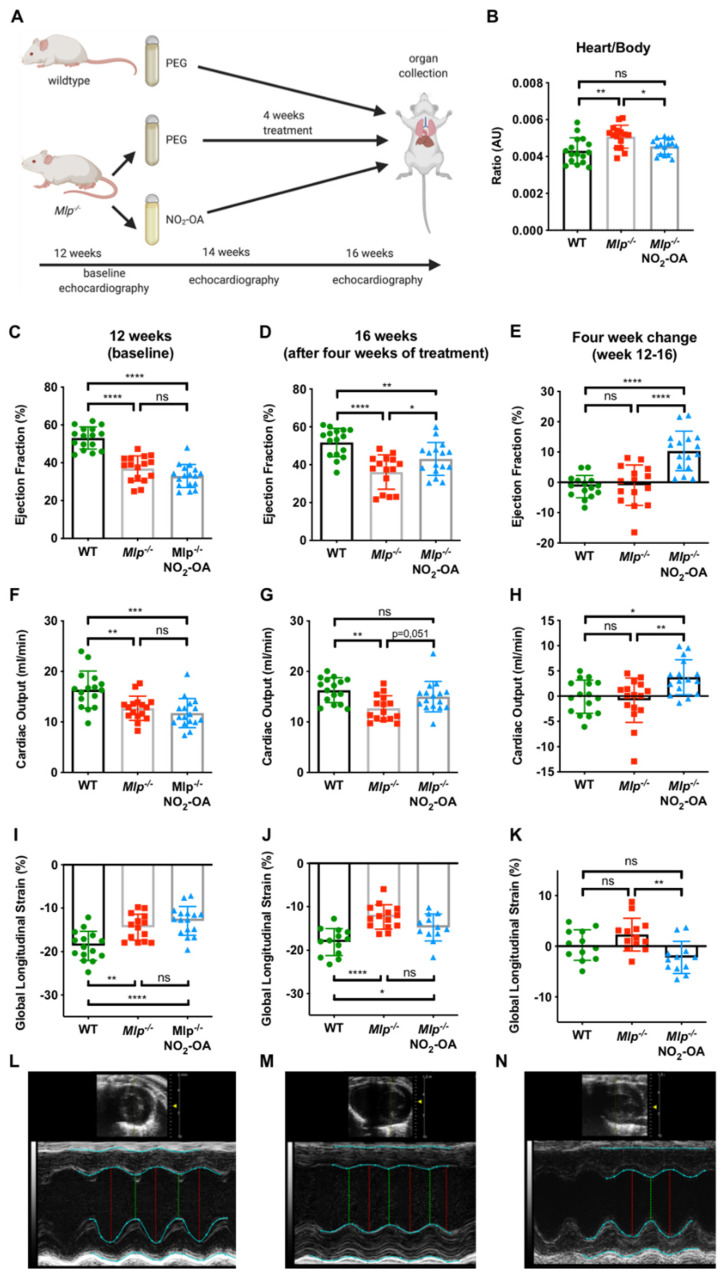
Effect of NO_2_-OA on left ventricular systolic function in DCM. (**A**) Experimental design of murine model treatment. (**B**) Heart-weight-to-body-weight ratio is reduced after 4 weeks of treatment with NO_2_-OA. Baseline echocardiographic assessment of (**C**) LVEF, (**F**) CO, and (**I**) GLS shows reduced baseline parameters in 12-week-old Mlp^−/−^ animals compared to WT and (**D**,**G**,**J**) improved parameters in 16-week-old animals after 4 weeks of treatment with NO_2_-OA. Absolute change of LVEF, CO, and GLS from weeks 12–16 is shown in (**E**,**H**,**K**). Representative M-mode echocardiography tracings of (**L**) WT, (**M**) Mlp^−/−^, and (**N**) Mlp^−/−^ + NO_2_-OA at 16 weeks. Data are expressed as mean ± SD, * *p* < 0.05, ** *p* < 0.01, *** *p* < 0.001, **** *p* < 0.0001, one-way ANOVA with Tukey multiple comparison test, *n* = 15–17 as indicated.

At the time of harvest (16 weeks of age, Figure 1A), the heart to body weight ratio was significantly greater in polyethylene glycol (PEG)-treated *Mlp^−/−^* mice compared to wildtype (WT) controls. However, the weight of the explanted hearts was significantly less in NO_2_-OA treated *Mlp^−/−^* animals, resulting in a heart to body weight ratio comparable to WT levels (Figure 1B and Figure S1). To evaluate potential cardioprotective properties of NO_2_-OA in DCM, we analyzed left ventricular (LV) systolic function. Baseline echocardiography at 12 weeks showed significantly reduced ejection fraction (EF), cardiac output (CO), and global longitudinal strain (GLS) in *Mlp^−/−^* mice, confirming the DCM phenotype of the model (Figure 1C,F,I). At week 14, NO_2_-OA treatment induced an absolute increase of 3.5% in LVEF and 2 mL/min in CO (Figure S1). Within four weeks, treatment with NO_2_-OA significantly improved EF, CO, and GLS in *Mlp^−/−^* mice, whereas these parameters remained unchanged (EF and CO) or were decreased further (GLS) in PEG-treated controls (Figure 1E,H,K). Morphologically, left ventricular end-diastolic volume was attenuated by NO_2_-OA in *Mlp^−/−^* mice (Figure S5). Treatment with NO_2_-OA did not affect LV function in WT mice, indicating an effect of NO_2_-OA under pathophysiological but not basal conditions (Figure S2).

### 2.2. NO_2_-OA Attenuates Myocardial Fibrosis in DCM

**Figure 2:**
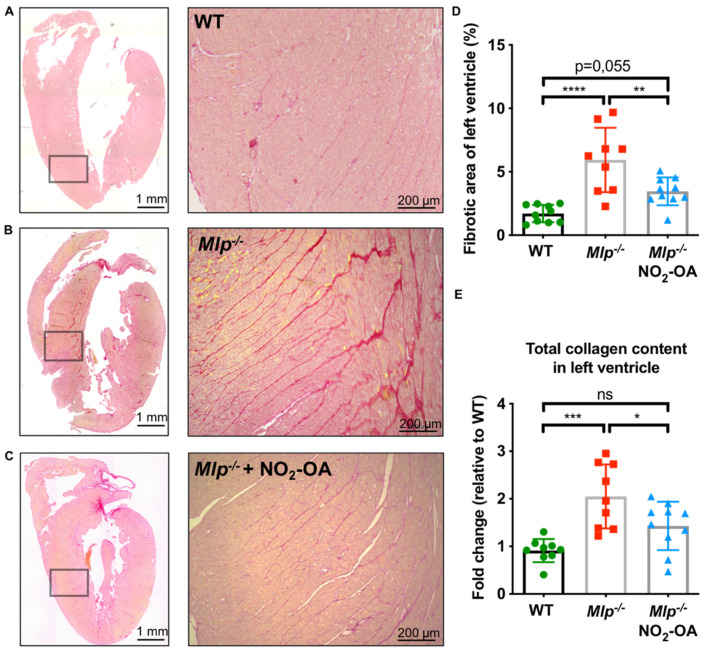
Effect of NO_2_-OA on interstitial myocardial fibrosis in DCM. (**A**–**C**) Representative images of whole heart sections and magnified areas from WT, Mlp^−/−^, and Mlp^−/−^ + NO_2_-OA stained with picrosirius red for collagen detection. (**D**) Analysis of fibrotic area shows significantly reduced interstitial fibrosis in mice treated with NO_2_-OA. (**E**) Biochemical analysis of total collagen content in LV tissue homogenates reveals a reduction of collagen by NO_2_-OA. Data are expressed as mean ± SD, * *p* < 0.05, ** *p* < 0.01, *** *p* < 0.001, **** *p* < 0.0001, one-way ANOVA with Tukey multiple comparison test, *n* = 9–10 as indicated, scalebar as indicated.

As myocardial fibrosis is a hallmark feature of DCM and anti-fibrotic properties of NO_2_-OA have been observed [14], we assessed the extent of left ventricular fibrosis via picrosirius red staining. As expected, extensive myocardial fibrosis was found in vehicle-treated Mlp^−/−^ mice compared to WT controls. Treatment with NO_2_-OA reduced the extent of LV myocardial fibrosis to WT levels (Figure 2A–D). Similarly, the total collagen content in LV homogenates was significantly increased in Mlp^−/−^ animals compared to WT controls and this effect was alleviated in Mlp^−/−^ mice treated with NO_2_-OA (Figure 2E). 

### 2.3. Mlp^−/−^ Mice Demonstrate Increased TGFβ Signaling

**Figure 3:**
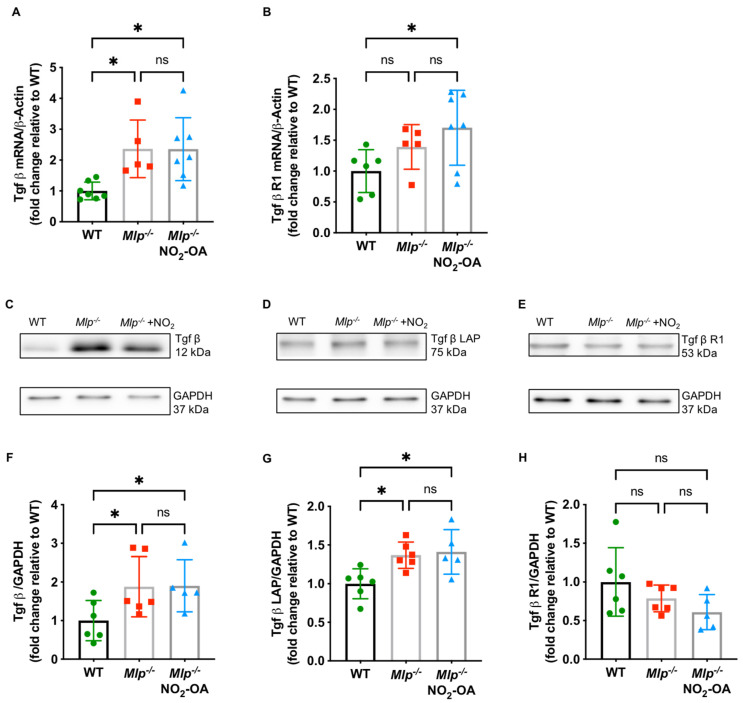
TGFβ signaling *Mlp^−/−^* hearts. (**A**) qPCR reveals increased mRNA expression levels of Tgfβ in LV of *Mlp^−/−^* mice, whereas (**B**) expression of Tgfβ R1 is unaffected. Representative immunoblot and quantification demonstrate increased protein expression levels of TGFβ (**C**,**F**) and TGFβ latency-associated protein (LAP) (**D**,**G**) in *Mlp*^−/−^ mice, whereas protein expression levels of TGFβ receptor 1 (R1) (**E**,**H**) is not influenced by the DCM phenotype. Data are expressed as mean ± SD, * *p* < 0.05, one-way ANOVA with Tukey multiple comparison test, *n* = 5–7 as indicated.

TGFβ is a key driver of myocardial fibrosis, with aberrant TGFβ signaling linked to pathogenic fibrosis. To test whether TGFβ signaling is altered in Mlp^−/−^ mice, we analyzed homogenized LV tissue by Immunoblot and qPCR and detected significantly increased protein and mRNA levels of TGFβ (Figure 3A,C,F) and the TGFβ latency-associated protein (LAP) (Figure 3B,D,G). NO_2_-OA treatment did not affect the expression of TGFβ and its receptor TGFβ-R1, suggesting a potential effect of NO_2_-OA downstream to the TGFβ signaling cascade (Figure 3E,H).

### 2.4. NO_2_-OA Modulates TGFβ Signaling in Isolated Primary Cardiac Fibroblasts

**Figure 4:**
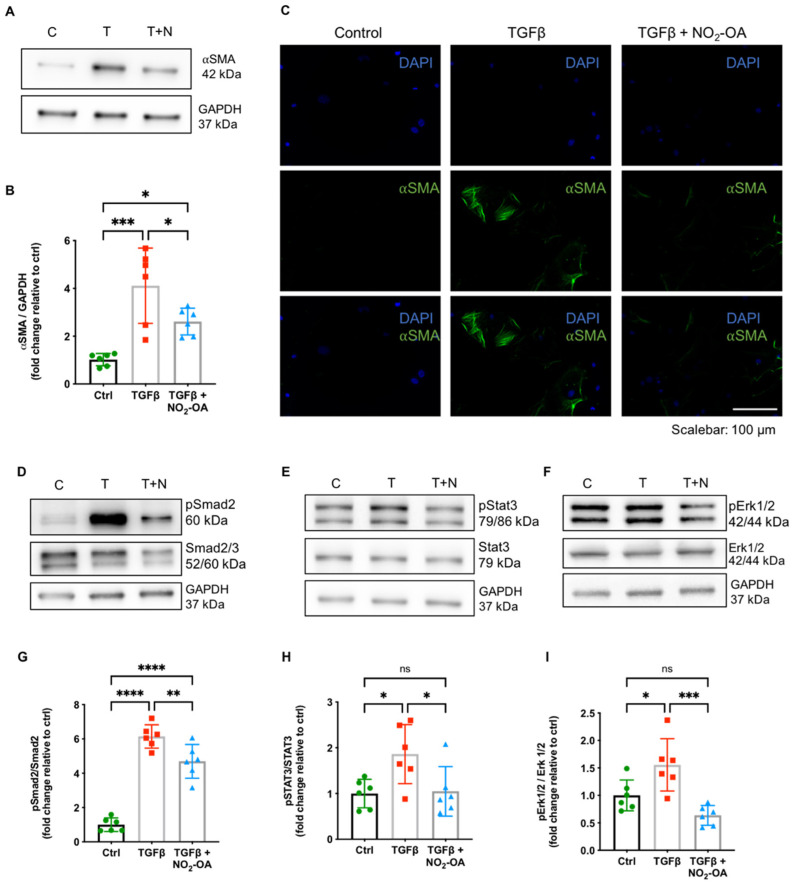
Effect of NO_2_-OA on TGFβ signaling in isolated primary cardiac fibroblasts. Representative (**A**) immunoblots, (**B**) quantification for expression of ⍺SMA, and (**C**) representative immunofluorescence show an increased ⍺SMA expression in cardiac fibroblasts upon treatment with TGFβ, which is significantly attenuated by concomitant treatment with NO_2_-OA. Protein expression analysis of the phosphorylation ratio of Smad2 (**D**,**G**), Stat3 (**E**,**H**), and Erk1/2 (**F**,**I**) revealed attenuated phosphorylation by NO_2_-OA. Data are expressed as mean ± SD, * *p* < 0.05, ** *p* < 0.01, *** *p* < 0.001, **** *p* < 0.0001, one-way ANOVA with Tukey multiple comparison test, *n* = 6 as indicated, scalebar as indicated.

NO_2_-OA attenuated myocardial fibrosis without affecting TGFβ levels in DCM. To mechanistically unravel the anti-fibrotic properties of NO_2_-OA, we investigated its effect on TGFβ downstream targets and fibrotic remodeling in vitro using primary isolated cardiac fibroblasts (CF). As expected, the stimulation of CF with TGFβ resulted in a significantly increased expression of the myofibroblast marker ⍺-smooth muscle actin (⍺SMA). Concomitant treatment with NO_2_-OA significantly attenuated ⍺SMA expression (Figure 4A–C). To test whether NO_2_-OA would affect CF activation by attenuated TGFβ signaling, we determined the extent of phosphorylation in canonical (small mothers against decapentaplegic (Smad2/3)) and non-canonical (signal transducer and activator of transcription 3 (Stat3) and extracellular signal-regulated kinase (Erk1/2)) downstream mediators of the TGFβ cascade. Concomitant treatment of CF with NO_2_-OA attenuated activation of both canonical and non-canonical TGFβ signaling (Figure 4D–I). Of note, fibroblasts of WT animals did not express MLP on the protein level (Figure S3).

## 3. Discussion

In the present study, we investigated the effects of NO_2_-OA on dilated cardiomyopathy in *Mlp*^−*/*−^ mice. We show for the first time that: (i) treatment with NO_2_-OA significantly improves left ventricular systolic function; (ii) the extensive myocardial fibrosis in *Mlp*^−*/*−^ mice is attenuated by NO_2_-OA; (iii) TGFβ signaling is increased in *Mlp*^−*/*−^ mice and (iv) NO_2_-OA prevents TGFβ-mediated transdifferentiation and phosphorylation of downstream targets in isolated primary cardiac fibroblasts.

DCM is characterized by left ventricular dilation and systolic dysfunction, which are both cardiac hallmarks of the *Mlp*^−*/*−^ mouse [23]. Guideline-directed medical therapy of DCM still mainly consists of neurohumoral intervention, i.e., Renin-Angiotensin-Aldosterone-System-Inhibition and beta-blockade [25,26]. A new treatment strategy for myocardial fibrosis is needed. Currently, anti-fibrotic therapy only consists of the small molecule drugs pirfenidone and nintedanib, which are used for the treatment of pulmonary fibrosis [27,28]. Both drugs only modestly slow lung function decline and are linked with dose-limiting toxicities. Here, we treated *Mlp*^−*/*−^ mice with NO_2_-OA, a member of the drug class of nitrated fatty acids. Treatment was started at 12 weeks of age, at which point, all mice had developed a clear DCM phenotype (Figure 1C,F,I). After four weeks of treatment, LVEF and CO had improved significantly, with the latter even returning to WT levels (Figure 1B,E). Of note, the heart rate was unchanged, indicating that the improved CO was driven by an enhanced stroke volume (Figure S4). EF and CO were unchanged in WT mice after four weeks of NO_2_-OA treatment and did not worsen in vehicle-treated *Mlp*^−*/*−^ mice (Figure S2), meaning that any improvement was exclusively seen in in *Mlp*^−*/*−^ mice receiving NO_2_-OA. The unaltered ED and CO in NO_2_-OA-treated WT mice is not surprising, given that NO_2_-OA is an endogenously produced signaling mediator and—like other NO_2_-FA—undergoes metabolism, nitroalkene inactivation by double bond reduction, and excretion within both biological and therapeutic levels [29]. The absence of cardiac or other measured phenotypical effects in healthy animals affirms the broad therapeutic range of NO_2_-OA in treating this and other cardiac fibrotic remodeling events and reveals encouraging off-target effect profiles. While EF and CO are widely accepted parameters for evaluation of left ventricular systolic function, speckle-tracking-based echocardiography provides refined analysis of myocardial dysfunction and is especially useful in detecting early changes in myocardial function [30,31,32]. Analysis of global longitudinal strain (GLS) revealed that vehicle-treated *Mlp*^−*/*−^ mice exhibit a spontaneous drop in myocardial function between the age of 12 and 16 weeks, further strengthening the beneficial effects of NO_2_-OA beyond EF and CO-improved GLS (Figure 1K).

While fibrotic remodeling is typically found in other cardiac pathologies, such as hypertensive heart disease or heart failure following myocardial infarction, increased levels of interstitial myocardial fibrosis are demonstrated in human DCM and are also key features in *Mlp*^−*/*−^ mice [23,33]. Herein, we show two- to three-fold increased interstitial collagen content compared to WT littermates and the degree of fibrotic area corresponds to what has been shown in human DCM patients [34]. Treatment with NO_2_-OA reduced the area of myocardial fibrosis and collagen content in *Mlp*^−*/*−^ mice to WT levels. Taken together, these results demonstrate an anti-fibrotic effect of NO_2_-OA in the setting of DCM. This observation is consistent with previous studies that report the alleviation of renal fibrosis and hypertension-induced atrial fibrotic remodeling by NO_2_-OA [14,35].

Various external stimuli including ischemia or chronic inflammation can induce fibrogenesis, as characterized by an excessive production of extracellular matrix proteins. As myocardial fibrosis was increased in *Mlp*^−*/*−^ mice and attenuated upon treatment with NO_2_-OA, we analyzed the expression of TGFβ, which represents the most common mediator for interstitial fibrosis in the heart and other organs such as the liver or kidneys [36,37]. *Mlp*^−*/*−^ mice showed increased levels of TGFβ mRNA and fibrosis-related protein expression, an event also observed in endomyocardial biopsies of DCM patients with a hypertensive, valvular, or ischemic etiology of heart failure. These DCM patients displayed greater TGFβ mRNA, collagen I and III protein expression compared to healthy subjects [33]. The fact that NO_2_-OA treatment neither influenced TGFβ nor TGFβ receptor 1 expression motivated the evaluation of effector mechanisms downstream of TGFβ itself. 

Phospho-immunoblotting of downstream effectors in the TGFβ signaling cascade revealed modulation of Smad2/3 and Erk1/2 in CF by NO_2_-OA. Phosphorylation of Smad2/3 is a hallmark of the canonical TGFβ signaling pathway. Attenuation of Smad2/3 phosphorylation and subsequently reduced atrial remodeling by NO_2_-OA has been demonstrated in 3T3 cells before. We could confirm this effect in left ventricular tissue and primary CF [14], both revealing biological relevance in living tissue and suggesting the primarily affected cell to be the CF. Smad-independent non-canonical TGFβ signaling includes the activation of Mitogen-activated kinases (MAPK) such as Erk1/2 [38,39]. NO_2_-OA suppresses phosphorylation of Erk1/2 in various cell types, including vascular smooth muscle cells in pulmonary hypertension, and peritoneal mesenchymal cells in a murine model of dialysis-related peritoneal damage [40,41]. Consequently, NO_2_-OA treatment prevented fibrotic remodeling in the right ventricle and the peritoneum, respectively. NO_2_-OA also inhibited phosphorylation of Stat3. This multi-faceted transcription factor, acting downstream of JAK activation, regulates intracellular processes such as inflammation, proliferation, and apoptosis [42]. Of relevance, Stat3 phosphorylation levels are increased in myocardial infarction-induced cardiac fibrosis and shRNA-mediated knockdown of its putative mediator EphrinB2 reduced the extent of cardiac fibrosis and improved LV systolic function [43]. In vitro, EphrinB2 affected Stat3 phosphorylation in cardiac fibroblasts; moreover, a critical crosstalk between Smad2/3 and Stat3 was seen in this setting [43]. Also, NO_2_-OA inhibits Stat3 phosphorylation via direct nitroalkylation in a human keratinocyte cell line [44]. Finally, only very recently, NO_2_-OA was shown to interfere with phosphorylation of Smad2/3, Stat3, and Erk1/2 in a murine model of Marfan’s disease, further supporting the data presented herein [45].

TGFβ stimulates a phenotypic conversion from resident CF to myofibroblasts, which are potent producers of extracellular matrix and major contributors to cardiac fibrosis, thereby inducing systolic dysfunction [46,47]. We show that NO_2_-OA effectively interferes with TGFβ-induced transdifferentiation of CF to myofibroblasts in vitro. After disruption of the TGFβ signaling cascade by the therapeutic agent, progression of fibrotic remodeling may have been stopped and existing myocardial fibrosis reversed. The enhancement of LV function is most likely a consequence of these long-term effects and would therefore occur with a temporal delay. Recent chemoproteomic profiling studies of NO_2_-OA targets showing an impact on proteins responsible for protein and lipid trafficking and turnover support these speculations [48,49].

This study has some limitations. *Mlp*^−*/*−^ mice serve as a classical model for heart failure, but interstitial myocardial fibrosis is not the exclusive or primary reason for disease onset. Future investigation of other animal models with robust fibrotic remodeling due to hypertensive heart disease induced by TAC- or angiotensin-/salt-induced systolic dysfunction would be revealing. Importantly, heart failure with preserved ejection fraction, for which new therapeutic options are desperately needed, is closely associated with myocardial fibrosis—thus the anti-fibrotic profile of NO_2_-OA may display benefit, especially since clinical trials using NO_2_-OA in patients have already been initiated. Moreover, investigating the effects of NO_2_-OA on *Mlp*^−*/*−^ cardiomyocytes could elucidate an additive effect of NO_2_-OA in the failing heart beyond attenuated CF activation, especially as beneficial properties of NO_2_-OA on ischemic heart failure in vivo and on malignant arrhythmias in isolated cardiomyocytes were previously shown [19,50]. Another area ripe for new drug candidate modeling is evaluating the reversal of established fibrosis, rather than the limitation of progressing fibrosis. We stress that specific effects of NO_2_-OA on CF in vitro cannot be defined as a specific mechanism or linear pathway modulation, as the broad reactivities and multiple targets of small molecule electrophiles result in pleiotropic responses. For example, further proteomic, genomic, and metabolomic studies can aid in better elucidating specific effectors in the NO_2_-FA modulation of TGFβ signaling and fibrogenic protein responses. Finally, NO_2_-FA can potentially act as NO-donors [20]; as such, afterload reduction must be discussed as a potential mechanism of action in DCM. However, as long-term clinical data on NO-donors in systolic heart failure is lacking, we did not further investigate this.

In summary, NO_2_-OA improves left ventricular function in *Mlp*^−*/*−^ mice by attenuating myocardial fibrosis. While increased levels of TGFβ in these animals are not influenced by this treatment, in vitro data from isolated primary cardiac fibroblasts reveal the inhibition of downstream effectors of the TGFβ signaling as a potential mechanism of action. Further studies are warranted to better understand the precise molecular actions of NO_2_-OA in the setting of dilated cardiomyopathy, especially as the small molecule nitroalkene has already proven safety in human phase I and phase 2 studies.

## 4. Materials and Methods

### 4.1. Animal Conditions and Experimental Design

All experimental procedures were performed with FVB/N mice. Csrp3/Mlp^−/−^ were kindly provided by R.K. These were held under clean laboratory conditions in NexGen™ Mouse 500 cages (Allentown Inc., Allentown, PA, USA) under a 12/12 h inverse light cycle, fed a standard rodent chow diet and water ad libitum. At 12 weeks of age, all animals were treated with vehicle polyethylene glycol/ethanol (90:10, *v*/*v*, PEG) or NO_2_-OA (1 nmol/(g * h)) via osmotic mini-pumps (ALZET Model 2002, Cupertino, CA, USA) that were subcutaneously implanted into the flanks. The total treatment duration was 4 weeks and pumps were changed once after 2 weeks. The specific regioisomer 10-nitro-octadec-9 (*E*)-enoic acid (NO_2_-OA) was provided by Bruce Freeman, Ph.D., University of Pittsburgh. All animal procedures were performed under isoflurane anesthesia and buprenorphine (0.01 mg/kg s.c.) was used as an analgesic before surgery and organ harvest. Animals were sacrificed by myocardial perfusion with saline and subsequent heart excision under deep anesthesia. All animal studies were approved by the local authorities (Landesamt für Natur, Umwelt und Verbraucherschutz NRW, Recklinghausen, 84-02.04.2015.A459) and by the University of Cologne Animal Care and Use Committees. Animal experiments conform to the guidelines from Directive 2010/63/EU of the European Parliament on the protection of animals used for scientific purposes. Observers were blinded for all quantitative analyses.

### 4.2. Echocardiography

Transthoracic echocardiography (TTE) was performed at 12, 14 and 16 weeks of age, i.e., one day before implantation of each osmotic minipump and at the end of the treatment period. Parasternal long axis view (PLAX) B-mode was performed to measure the following parameters: Ejection fraction (EF) and global longitudinal strain (GLS) using a modified Simpson’s monoplane disk technique was assessed in B-mode. Cardiac output was calculated (stroke volume * heart rate). A Vevo 3100 ultrasound imaging system and a MX550D transducer were used for echocardiography and images were analyzed with VevoLab Software (all Fujifilm VisualSonics, Toronto, ON, Canada).

### 4.3. Histology and Total Collagen Assay

Harvested LVs were fixed in 4% paraformaldehyde overnight at 4 °C and subsequently embedded in paraffin. Histological sections of 4 µm thickness were stained in picrosirius red solution. Slides were then photographed in 20× magnification in brightfield using a Keyence BZ-9000E microscope (Keyence, Osaka, Japan). The extent of fibrosis was calculated as the area of picrosirius red signal in relation to total tissue area by color thresholding using the Keyence BZ2-Analyser Software.

Left ventricular collagen content was measured using the Abcam total collagen assay kit (ab222942, Abcam, Cambridge, United Kingdom) according to manufacturer’s protocol. Briefly, 10 µg of freshly harvested left ventricles were homogenized in aqua dest. using a Peqlab Tissue Lyser (VWR, Radnor, PA, USA, #000-158) followed by an alkaline hydrolysis to yield free hydroxyproline, which was subsequently oxidized yielding chromophore that was detected using a spectrophotmeter (Thermo Fisher Scientific, Waltham, MA, USA, Type 357, #357-902221T) at OD 560 nm.

### 4.4. Isolation, Cultivation and Stimulation of Cardiac Fibroblasts

Hearts were harvested from 12-week-old WT mice. The remaining blood was removed mechanically in sterile ice-cold PBS. Ventricles were cut into small pieces (1–2 mm^2^) and digested in a semi-automated dissociation process using the GentleMACS Dissociator and Multi Tissue Dissosciation Kit 2 (Miltenyi Biotec, Bergisch-Gladbach, Germany) according to the manufacturer’s protocol. The single-cell suspension was then resuspended in standard cultivation medium (DMEM+ Glutamax, PAN-Biotech, Aidenbach, Germany) containing 10% fetal bovine serum (FBS) and 1% Penicillin/Streptomycin. Additionally, medium was supported with 0.1% Fibroblast Growth Factor (Recombinant Human FGF-basic (154 a.a.) Peprotech, Rocky Hill, NJ, USA). Cells were seeded in 6-well plates precoated with 1% gelatin at 37 °C and 5% CO_2_. Isolated cells were adherent and morphologically compatible with fibroblasts. Cells were split at 70–80% confluency and used for analysis after 3rd passage. Prior to stimulation, fibroblasts were placed in starving medium containing 0.1% FBS for 18 h. Cells were then stimulated with TGFβ with a concentration of 10 ng/mL (premium-grade recombinant human TGFβ1, Miltenyi Biotec, Bergisch-Gladbach, Germany), 1 µM NO_2_-OA, or both. Cells were harvested after 6 and 24 h of treatment.

### 4.5. Quantitative Real-Time PCR

RNA from cell culture and left-ventricular tissue was isolated using the Direct-zol RNA Microprep (Zymo Research, Freiburg, Germany) and reverse transcribed to cDNA using a high-capacity cDNA reverse transcription kit (Thermo Fisher Scientific, Waltham, MA, USA). Gene expression was examined using GoTaq® Master Mix (Promega, Walldorf, Germany) in quantitative real-time PCR (Applied Biosystems 7300 cycler, Thermo Fisher Scientific, Waltham, MA, USA). Primer sequences are provided in Table S1. The levels of mRNA expression were determined by normalizing all data to β-actin using the 2−ΔΔCt method. Normalization was performed by using samples of either homogenized tissue from wild type animals or untreated cells, respectively.

### 4.6. Immunoblot

Both left-ventricular tissue and cell lysates were resuspended in radioimmunoprecipitation assay buffer with 1xPhosSTOP™ (Sigma-Aldrich, St. Louis, MO, USA) und 1× complete ultra-mini EDTA-free (Sigma-Aldrich, St. Louis, MO, USA), supplemented with 4×Tris-Glycin-Buffer. To separate proteins by their molecular weight, equal amounts (7.5–15 µg) of protein were loaded to 12% SDS-PAGE. Afterwards, samples were transferred to a nitrocellulose membrane (VWR, Darmstadt, Germany) and blocked with 5% bovine serum albumin. Membranes were incubated with primary antibodies against *α*-smooth muscle actin (Abcam ab5694, 1:5000, Abcam, Cambridge, UK), Smad 2/3 (Cell Signalling, Danvers, MA, USA, #8685, 1:1000, Cell Signaling Technology, Danvers, MA, USA), phospho-Smad 2 (Ser465/467, Sigma #3849-I, 1:1000, Sigma Aldrich, St. Louis, MO, USA), Stat3 (#30835, 1:1000), phospho-Stat3 (Tyr705, #9145, 1:1000), p44/42 MAPK (Erk1/2, #4695, 1:1000) and phospho-p44/42 MAPK (Erk1/2, Thr202/Tyr204, #9101, 1:1000, all purchased from Cell Signaling Technology, Danvers, MA, USA) at 4 °C overnight. Secondary antibody (Anti-Rabbit IgG Peroxidase antibody, Sigma Aldrich, #A0545, 1:10,000) was applied to all membranes. Signal was detected by using SuperSignal™ West Femto Maximum Sensitivity Substrate (Thermo Fisher Scientific, Waltham, MA, USA) and ECL™ Start immunoblotting Detection Reagent (GE Healthcare, Chicago, IL, USA). Protein levels were quantified according to optical density (OD) using FusionCapt Advance Software (Vilber Smart Imaging, Collégien, France. Phospho-signal was related to signal of unphosphorylated expression levels, after stripping the membrane in 5 M NaOH for approximately 5 min depending on band intensity. GAPDH (Cell Signaling, Danvers, MA, USA, #2118l, 1:7500) served as loading control in all other immunoblots as indicated.

### 4.7. Immunofluorescene Staining

Cells were seeded in 8-well chamber slides (Thermo Fisher Scientific, Lab-Tek II Chamber Slide, System 154534, New York, NY, USA). Stimulation was performed as described and cells were fixed in 100% cold ethanol for ten minutes followed by 1% of formaldehyde overnight. Cells were permeabilized with 0.1% of Triton in PBS, blocked in 1% BSA, and stained with aSMA (Abcam, Anti-alpha smooth muscle Actin antibody, #ab5694) at 4 °C overnight. After staining, cells were washed in PBS and incubated with an Alexa Fluor 588 secondary antibody (Thermofisher Scientific, Goat anti-rabbit, #A11008) for one hour at room temperature. Cell nuclei were stained with DAPI. Slides were mounted with fluorescence mounting medium (Agilent Dako, Fluorescence Mounting Medium, # S302380-2).

### 4.8. Statistical Analysis

Data are displayed as mean ± SD. Shapiro–Wilk test was utilized to test for normal distribution. Statistical differences were analyzed using the ordinary one-way analysis of variance (ANOVA) followed by the Tukey adjustment for post hoc multiple comparison, as indicated in the legend of each figure. A *p*-value of less than 0.05 was considered to be statistically significant.

## Supplementary Materials

The following are available online at https://www.mdpi.com/article/10.3390/ijms22169052/s1.
