# Supplementary material for: Nitro-Oleic Acid (NO2-OA) Improves Systolic Function in Dilated Cardiomyopathy by Attenuating Myocardial Fibrosis
Source: Int J Mol Sci. 2021 Aug 22;22(16):9052. doi: 10.3390/ijms22169052 (PMC8396484; doi:10.3390/ijms22169052)
Supplement: Supplementary file 1 [file ijms-22-09052-s001.zip › ijms-1351984-supplementary.pdf]

Nitro-oleic acid (NO_2-OA) improves systolic function in dilated cardiomyopathy by attenuating myocardial fibrosis

Supplemental Material

Supplemental Figure S1

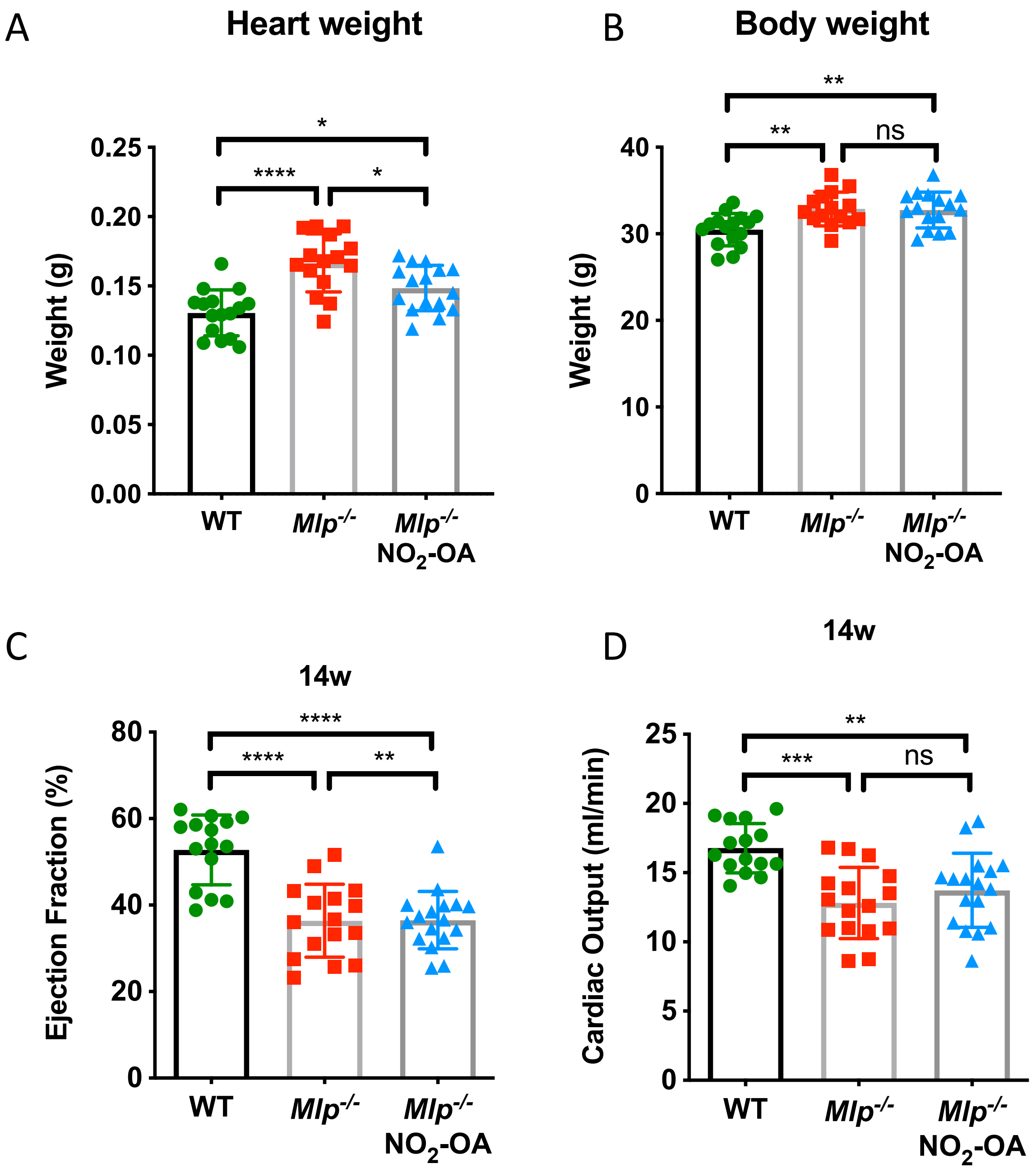

Supplemental Figure S2

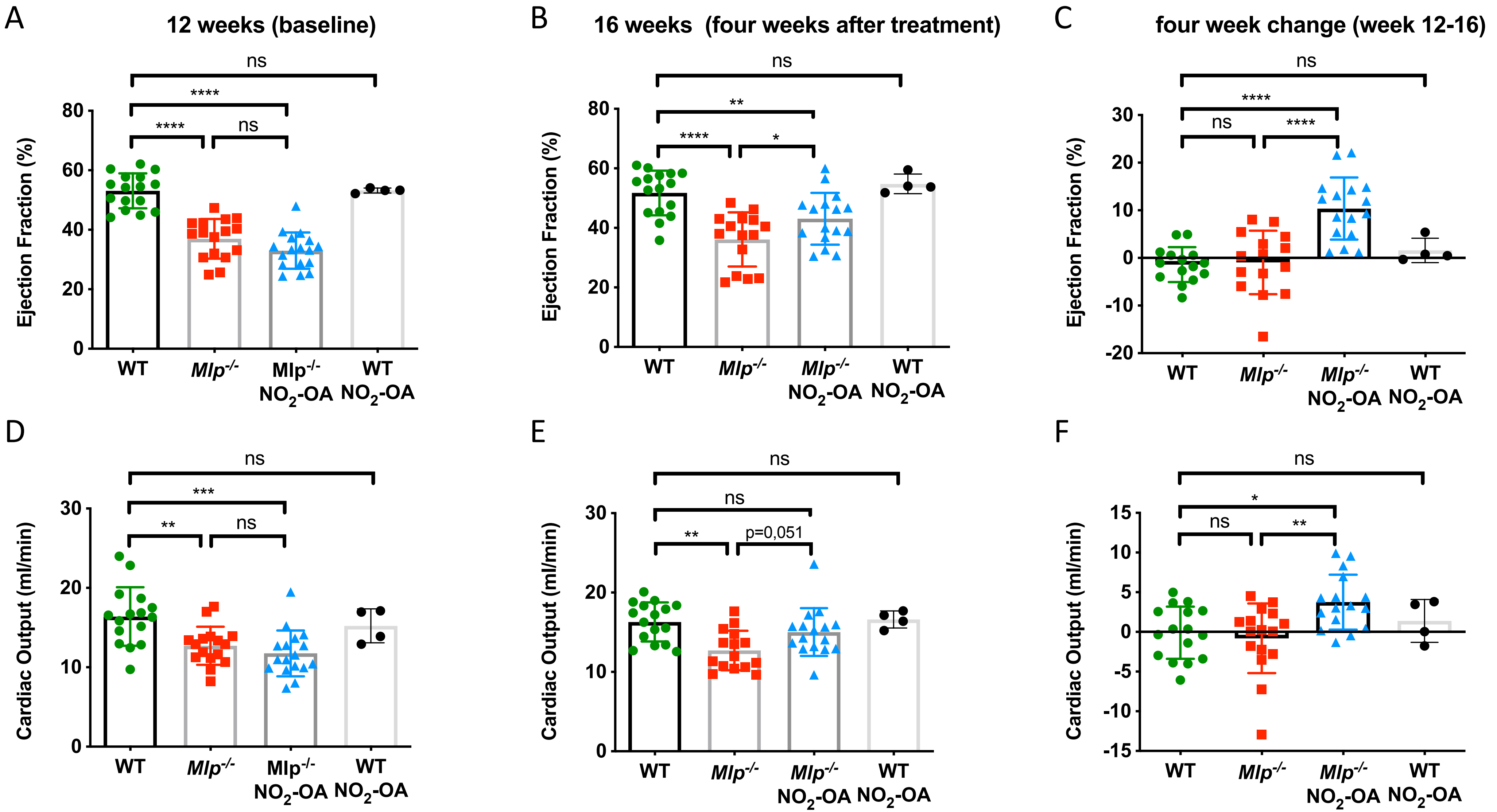

Supplemental Figure S3

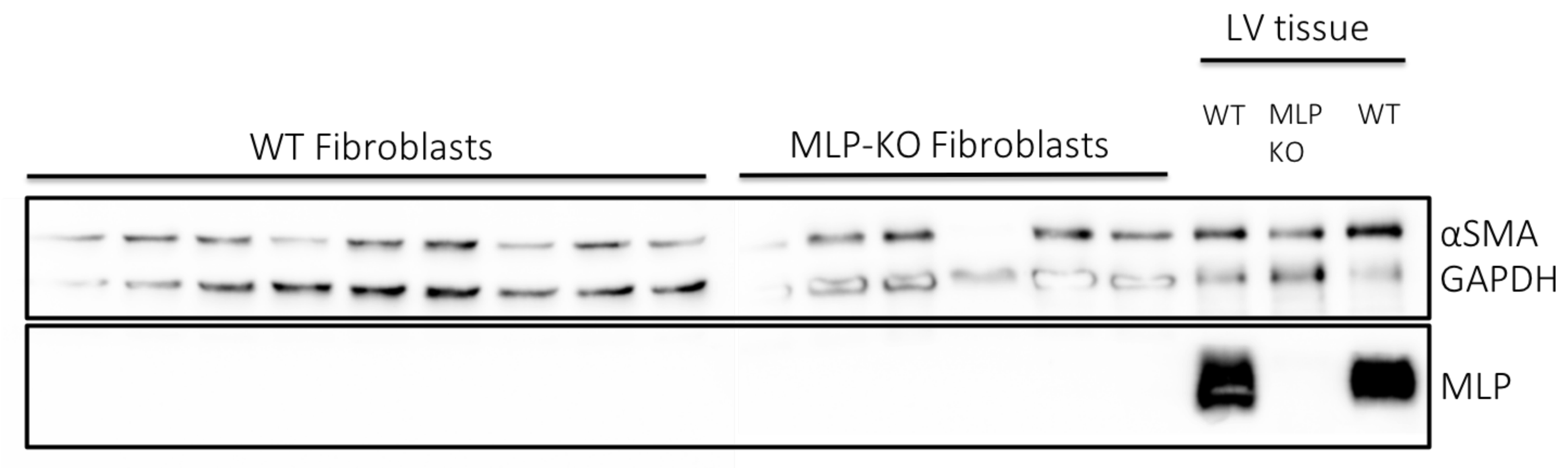

Supplemental Figure S4

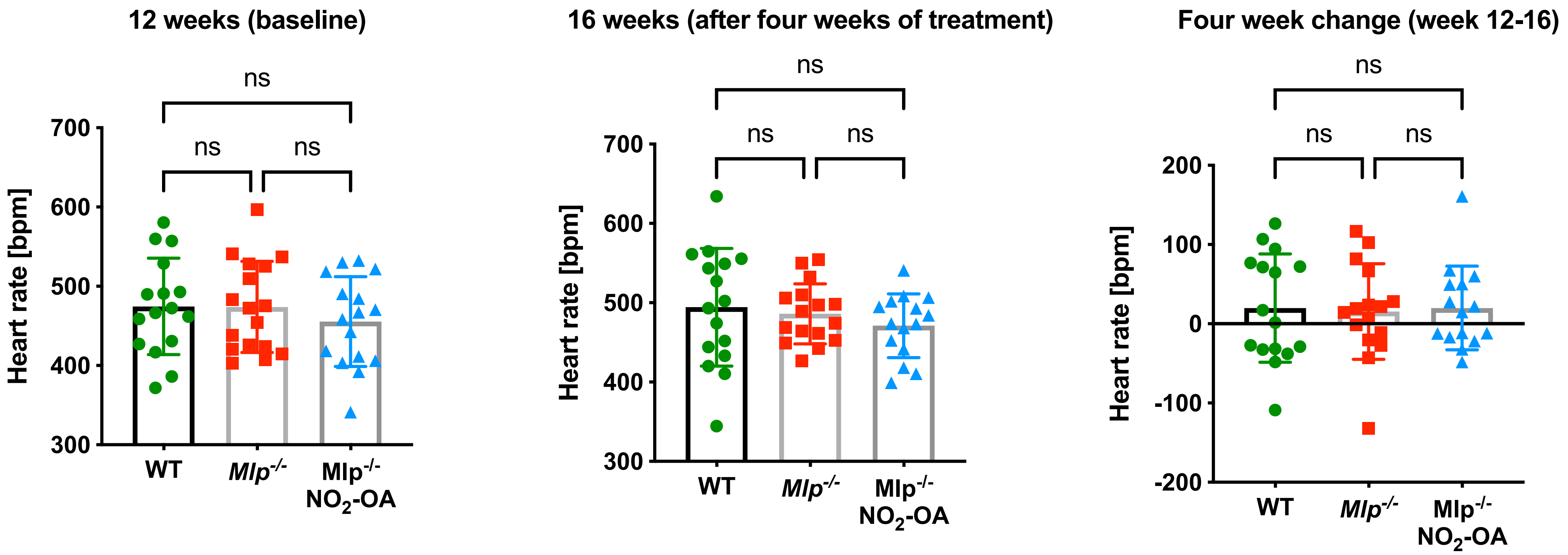

Supplemental Figure S5

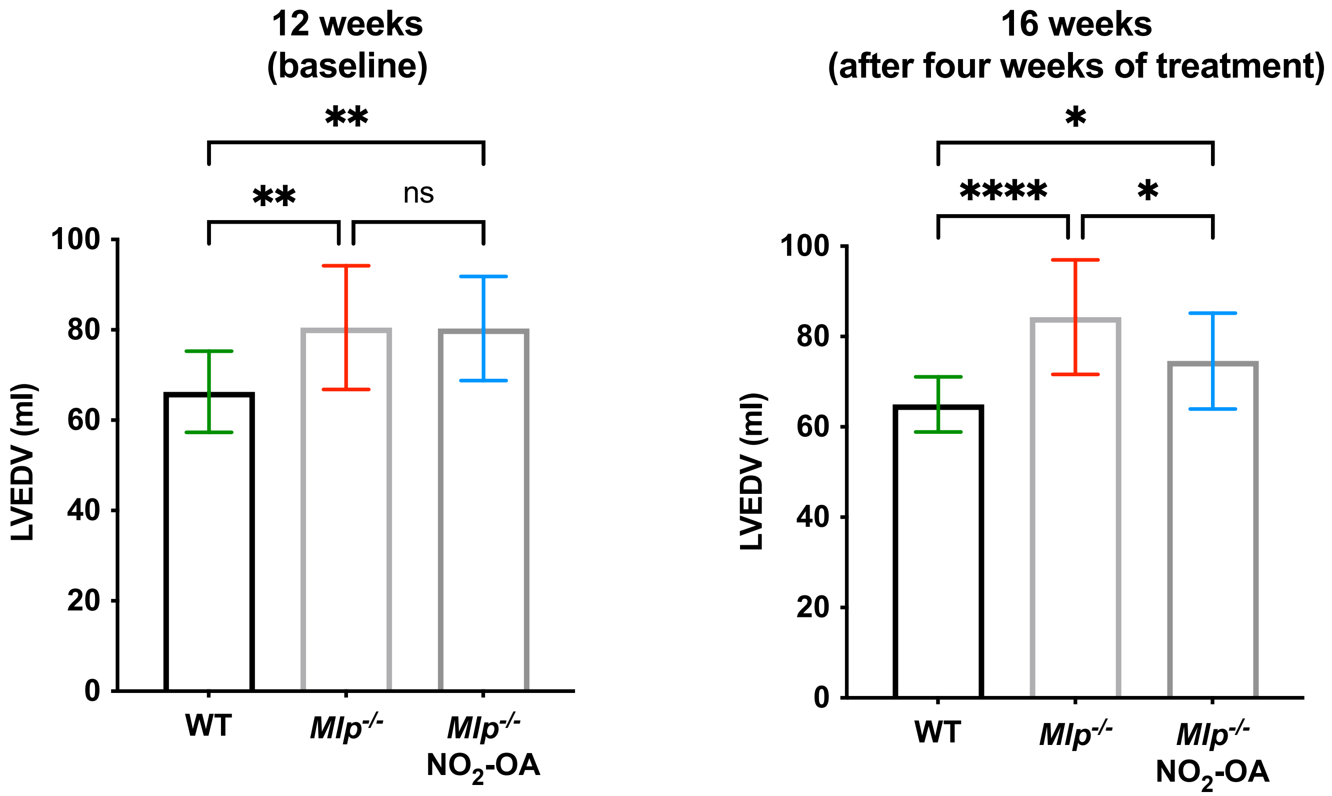

Uncropped Western Blot – as used in Figure 3

C= unstimulated control
T= stimulated with TGFb 10ng/ml
TN= TGFb + NO_2OA

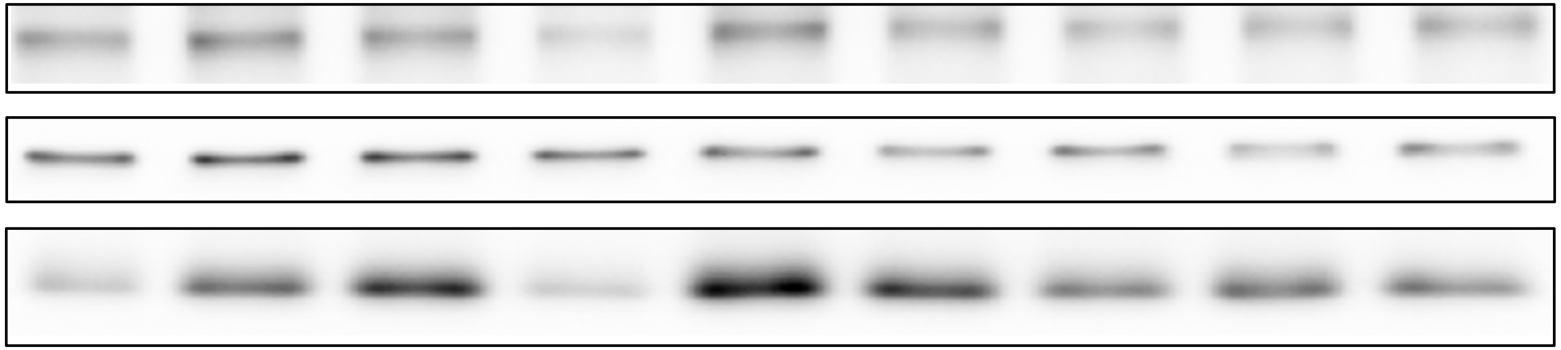

Conditions:
12,5 µg sample / pocket on 12% Gel
Blot on NC at 200 mA for 2 h
Block 1h in 5% BSA

1st AB:
aSMA 1:5000 at 4°C o/n

2nd AB: anti-Rb
In 5% BSA at RT for 2h

Detection: ECL/Femto 4 to 1
Detection time aSMA 10 s

Uncropped Western Blot – Focus on aSMA (as used in Figure 4A)

C= unstimulated control
T= stimulated with TGFb 10ng/ml
TN= TGFb + NO_2OA

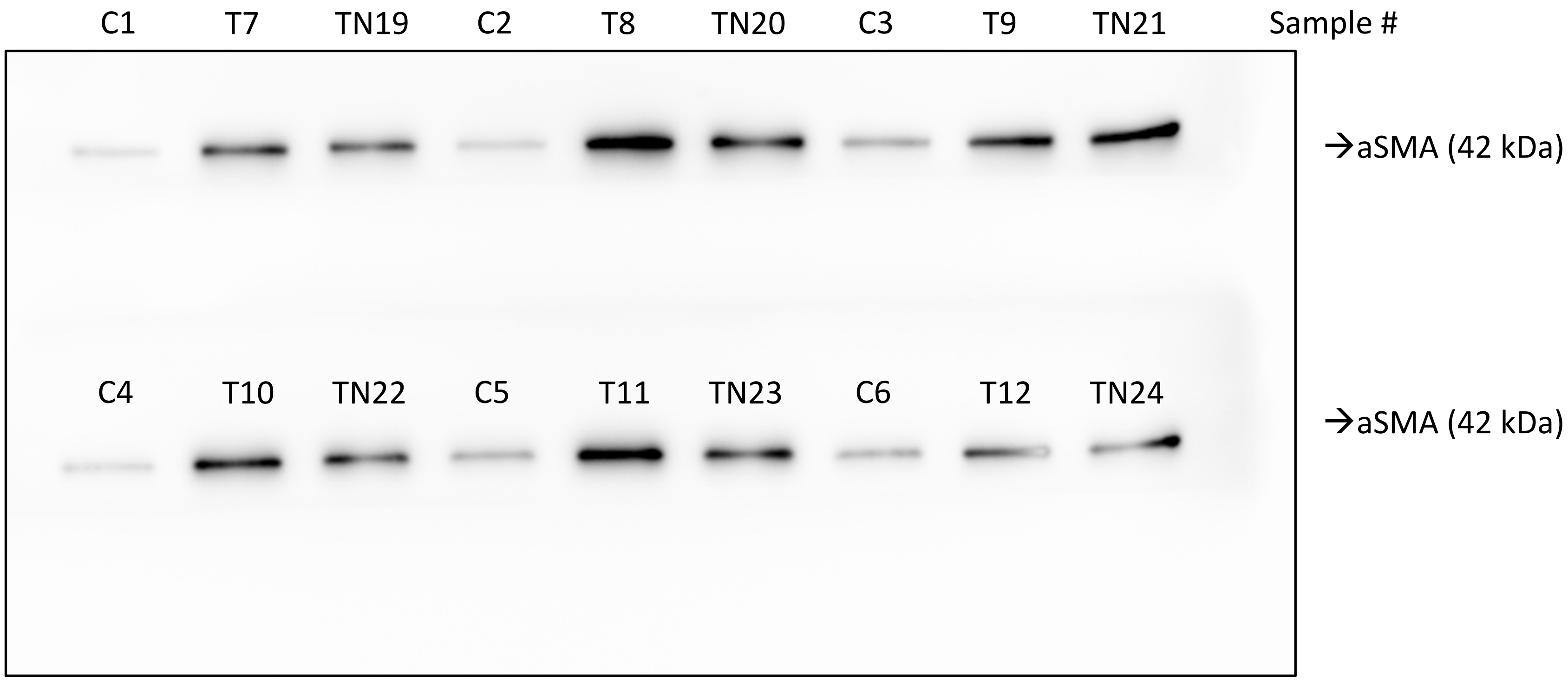

<u>**Conditions**</u>:
12,5 µg sample / pocket on 12% Gel
Blot on NC at 200 mA for 2 h
Block 1h in 5% BSA

1st AB:
aSMA 1:5000 at 4°C o/n

2nd AB: anti-Rb
In 5% BSA at RT for 2h

Detection: ECL/Femto 4 to 1
Detection time aSMA 10 s

Uncropped Western Blot – Focus on GAPDH (as used in Figure 4A)

C= unstimulated control
T= stimulated with TGFb 10ng/ml
TN= TGFb + NO_2OA

C1 T7 TN19 C2 T8 TN20 C3 T9 TN21 Sample #

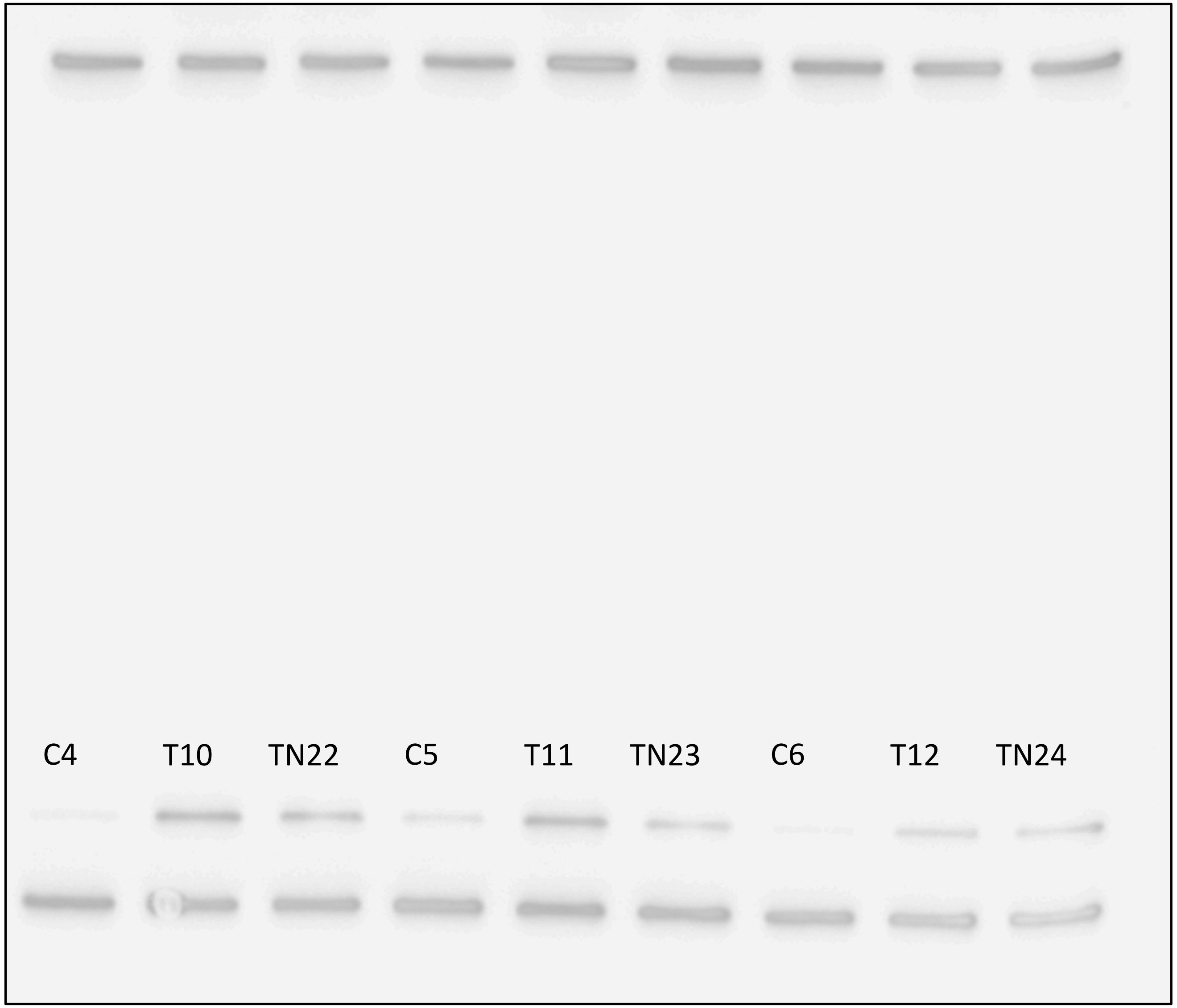

→GAPDH (37 kDa)

→aSMA (42 kDa) see previous slide

→GAPDH (37 kDa)

<u>**Conditions**</u>:
12,5 µg sample / pocket on 12% Gel
Blot on NC at 200 mA for 2 h
Block 1h in 5% BSA

1st AB:
GAPDH 1:7500 at 4°C o/n

2nd AB: anti-Rb
In 5% BSA at RT for 2h

Detection: ECL/Femto 4 to 1
Detection time GAPDH <1 s

Uncropped Western Blot – Focus on pSmad 2 pSer 465/467 (as used in Figure 4C)

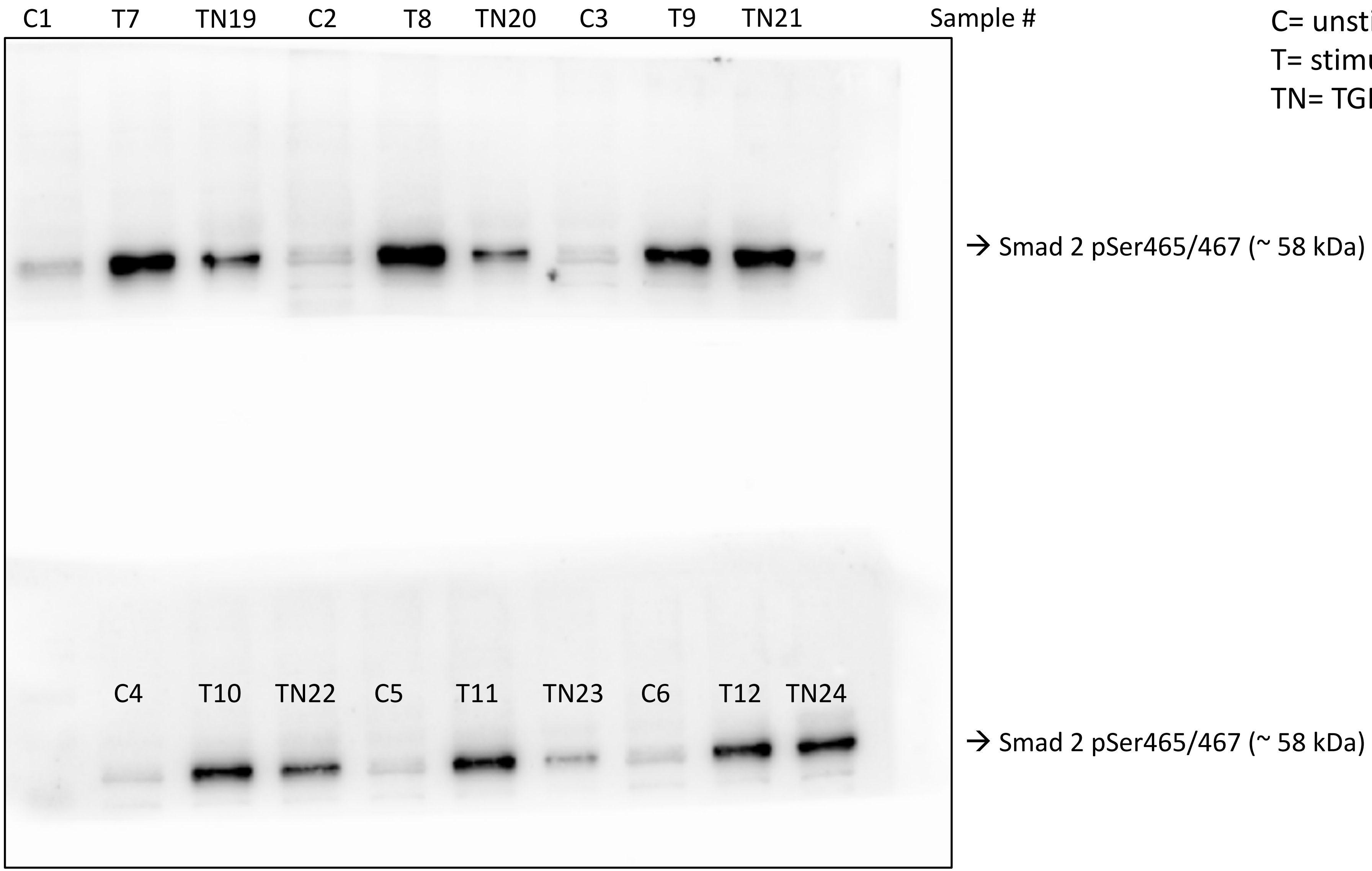

C= unstimulated control
T= stimulated with TGFb 10ng/ml
TN= TGFb + NO_2OA

<u>Conditions</u>:
12,5 µg sample / pocket on 12% Gel
Blot on NC at 200 mA for 2 h
Block 1h in 5% BSA

1st AB:
Smad 2 pSer465/467 1:1000 at 4°C o/n

2nd AB: anti-Rb
In 5% BSA at RT for 2h

Detection: ECL/Femto 4 to 1
Detection time Smad 2 pSer465/467 30 s

C= unstimulated control
T= stimulated with TGFb 10ng/ml
TN= TGFb + NO_2OA

Uncropped Western Blot – Focus on Total Smad 2/3 (as used in Figure 4C)

C1 T7 TN19 C2 T8 TN20 C3 T9 TN21

Sample #

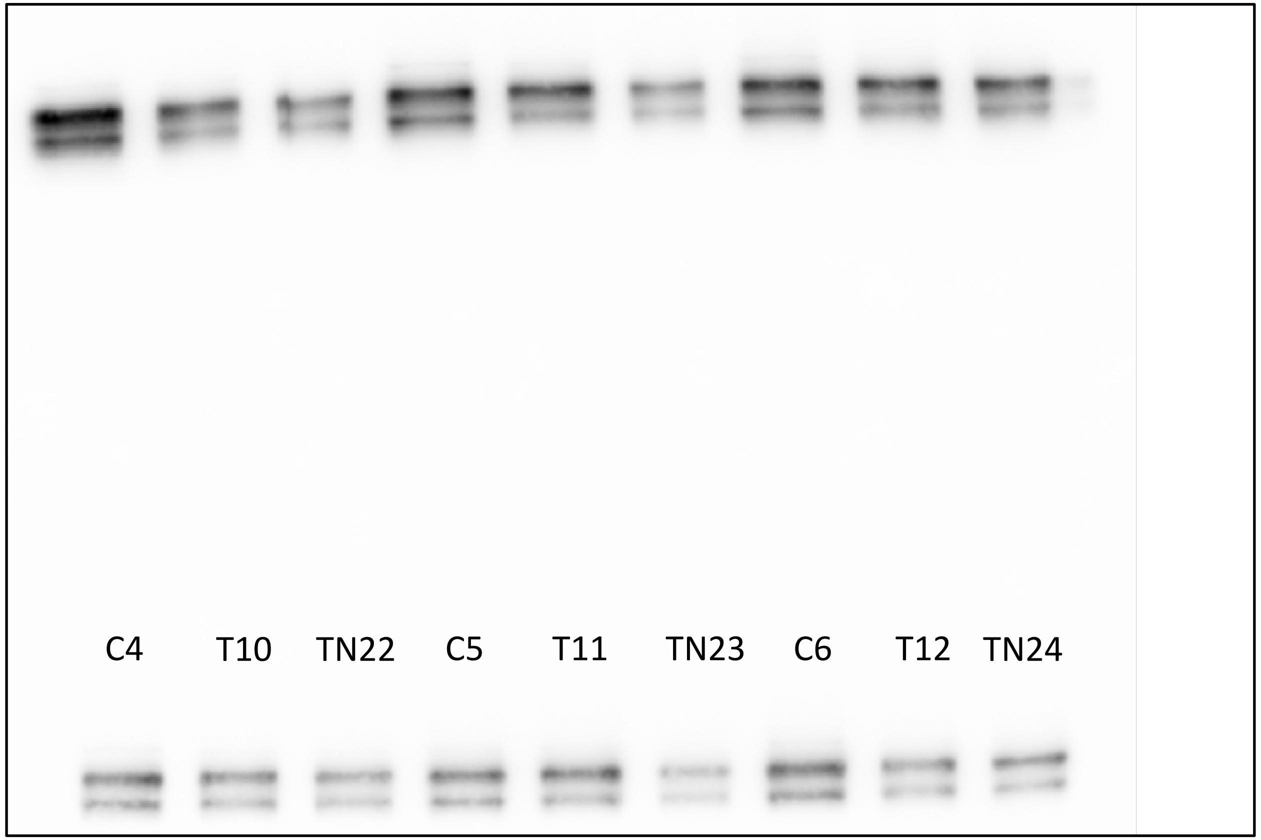

→Total Smad2/3 (52/60 kDa)

→Total Smad2/3 (52/60 kDa)

<u>**Conditions**</u>:
12,5 µg sample / pocket on 12% Gel
Blot on NC at 200 mA for 2 h
Block 1h in 5% BSA

1st AB:
After stripping with 5M NaOH for 5 min
Total Smad 2/3 1:1000 at 4°C o/n

2nd AB: anti-Rb
In 5% BSA at RT for 2h

Detection: ECL/Femto 2 to 1
Detection time Smad 2/3 60s

Uncropped Western Blot – Focus on GAPDH (as used in Figure 4C)

C= unstimulated control
T= stimulated with TGFb 10ng/ml
TN= TGFb + NO_2OA

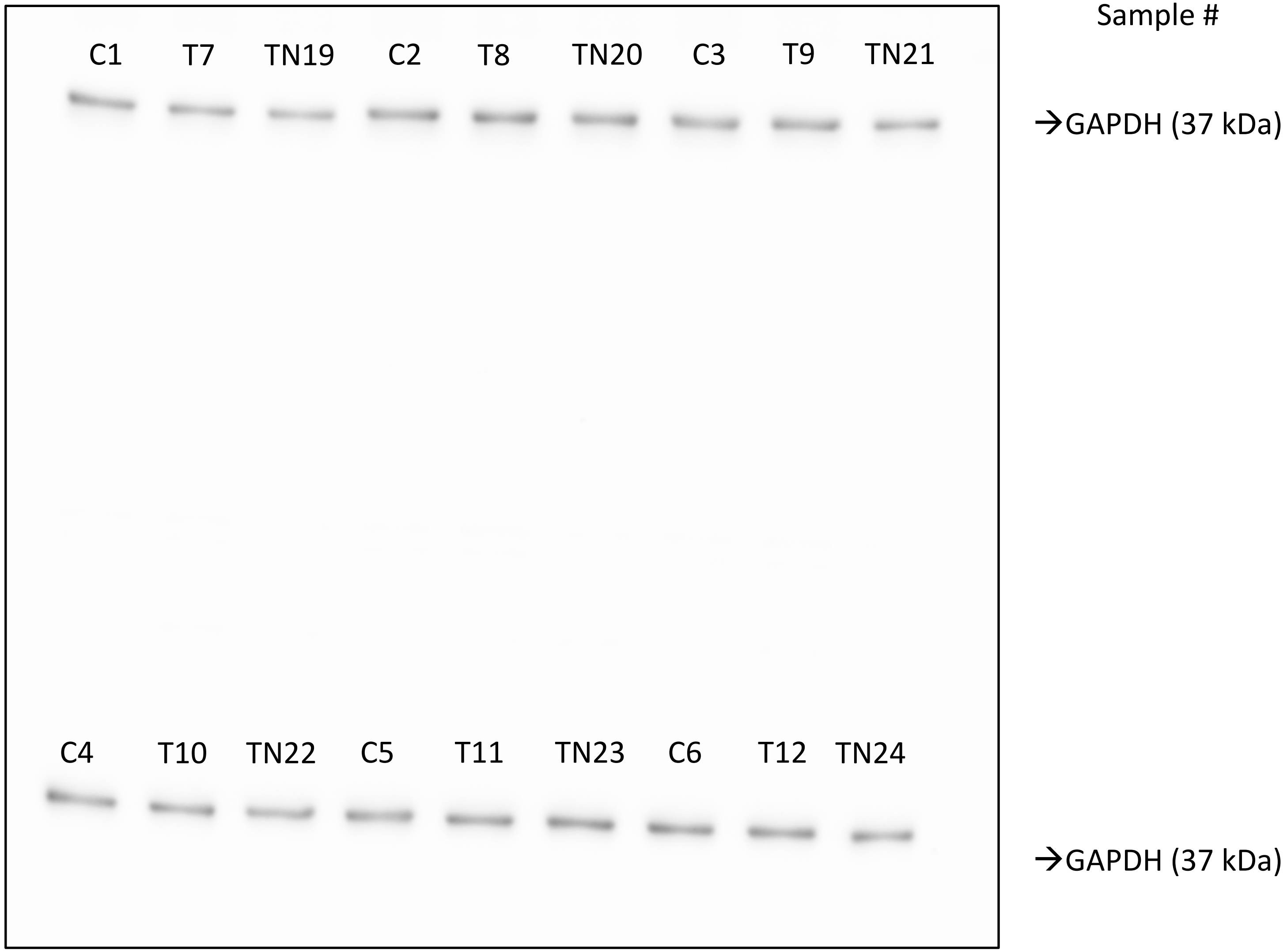

Conditions:
12,5 µg sample / pocket on 12% Gel
Blot on NC at 200 mA for 2 h
Block 1h in 5% BSA

1st AB:
GAPDH 1:7500 at 4°C o/n

2nd AB: anti-Rb
In 5% BSA at RT for 2h

Detection: ECL/Femto 4 to 1
Detection time GAPDH 1,5 s

C= unstimulated control
T= stimulated with TGFb 10ng/ml
TN= TGFb + NO_2OA

Uncropped Western Blot – Focus on STAT3 (as used in Figure 4D)

C1 T7 TN19 C2 T8 TN20 C3 T9 TN21 Marker Sample #

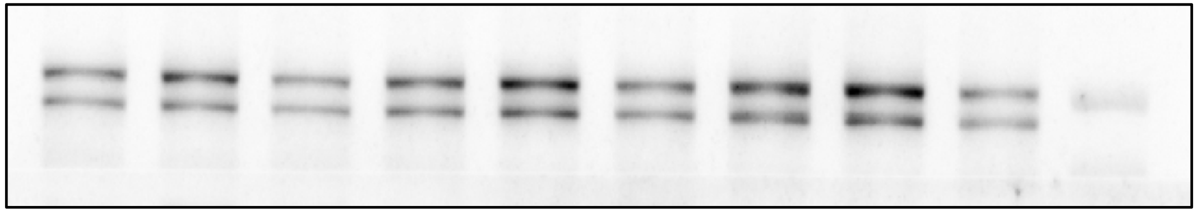

→Total Stat3 (79 kDa)

C4 T10 TN22 C5 T11 TN23 C6 T12 TN24

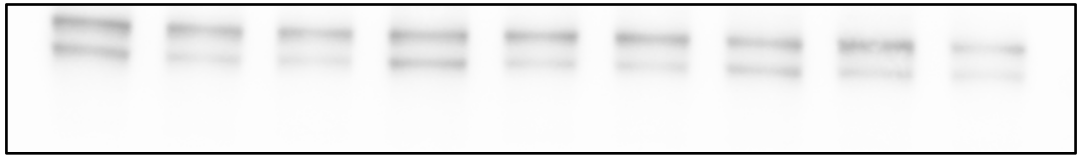

→Total Stat3 (79 kDa)

Conditions:
12,5 µg sample / pocket on 12% Gel
Blot on NC at 200 mA for 2 h
Block 1h in 5% BSA

1st AB:
After stripping with 5M NaOH for 5 min
Total Stat3 1:1000 at 4°C o/n

2nd AB: anti-Rb
In 5% BSA at RT for 2h

Detection: ECL/Femto 2 to 1
Detection time total Stat 3 60 s

Uncropped Western Blot – Focus on STAT3 (as used in Figure 4D)

C= unstimulated control
T= stimulated with TGFb 10ng/ml
TN= TGFb + NO_2OA

C1 T7 TN19 C2 T8 TN20 C3 T9 TN21 Sample #

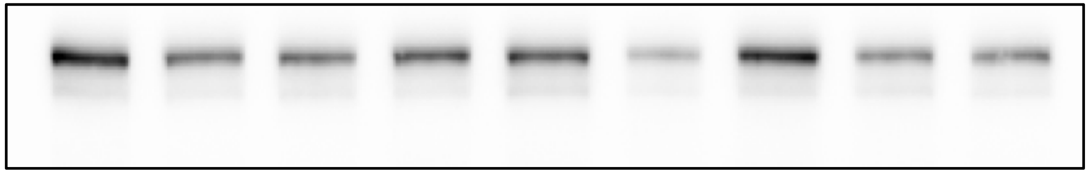

→Stat3 pTyr705 (86 kDa)

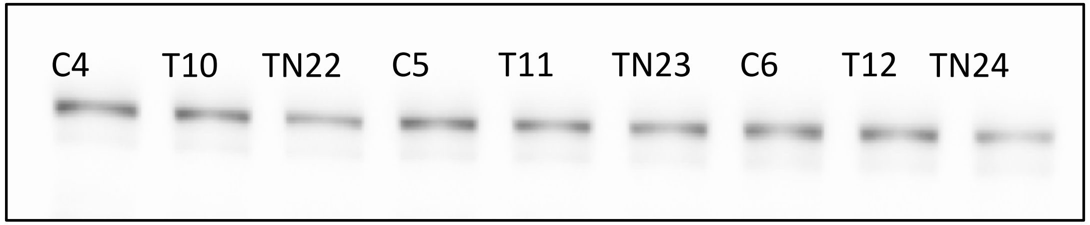

→Stat3 pTyr705 (86 kDa)

<u>Conditions</u>:
12,5 µg sample / pocket on 12% Gel
Blot on NC at 200 mA for 2 h
Block 1h in 5% BSA

1st AB:
Stat3 pTyr705 1:1000 at 4°C o/n

2nd AB: anti-
In 5% BSA at RT for 2h

Detection: ECL/Femto 1 to 1
Detection time Stat3 pTyr705 30 s

Uncropped Western Blot – Focus on Erk 1/2 pThr202/Tyr204 (as used in Figure 4E)

C= unstimulated control
T= stimulated with TGFb 10ng/ml
TN= TGFb + NO_2OA

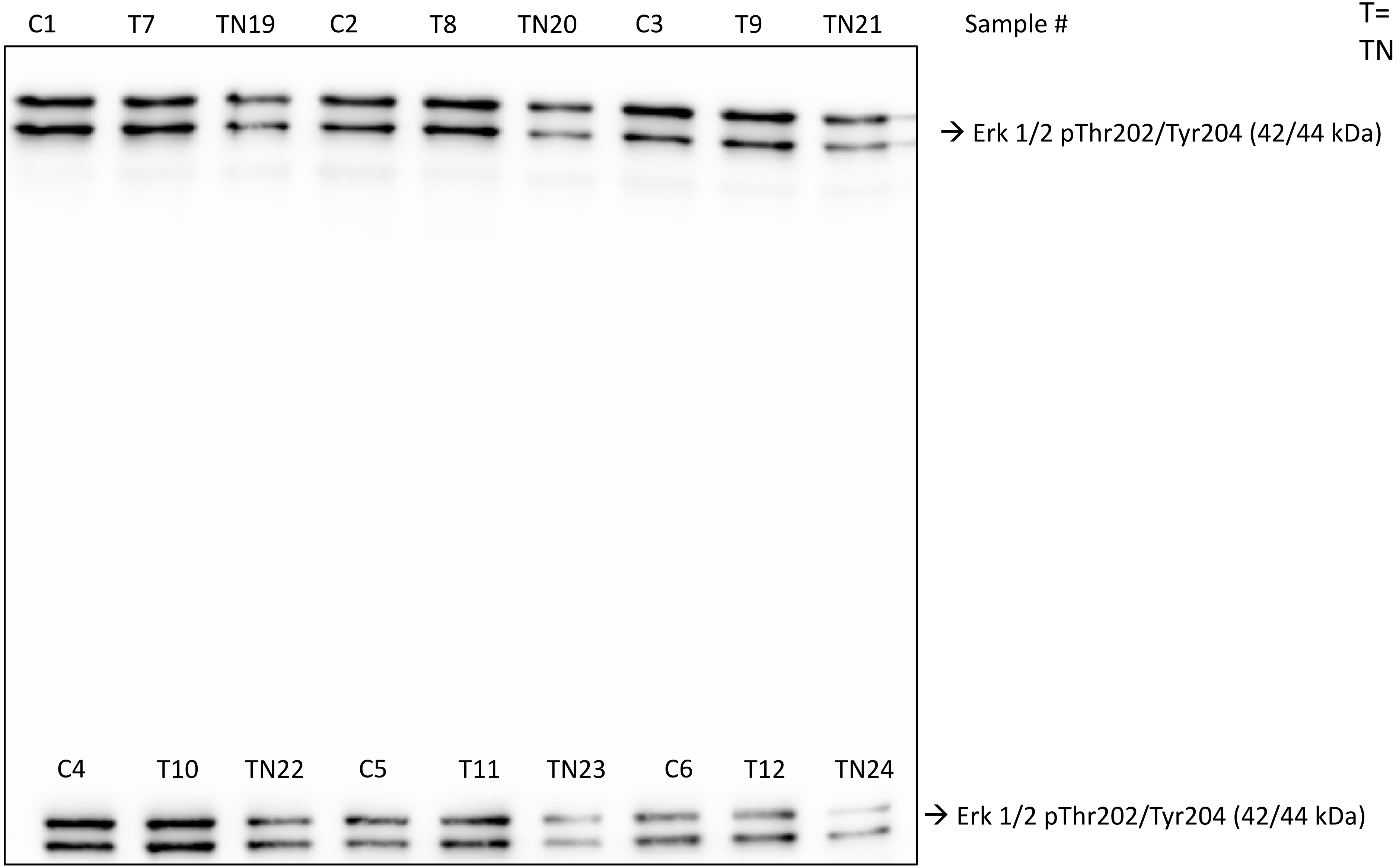

<u>**Conditions**</u>:
12,5 µg sample / pocket on 12% Gel
Blot on NC at 200 mA for 2 h
Block 1h in 5% BSA

1st AB:
Erk 1/2 pThr202/Tyr204
1:1000 at 4°C o/n

2nd AB: anti-Rb
In 5% BSA at RT for 2h

Detection: ECL/Femto 4 to 1
Detection time aSMA 10 s

Uncropped Western Blot – Focus on Total Erk 1/2 (as used in Figure 4E)

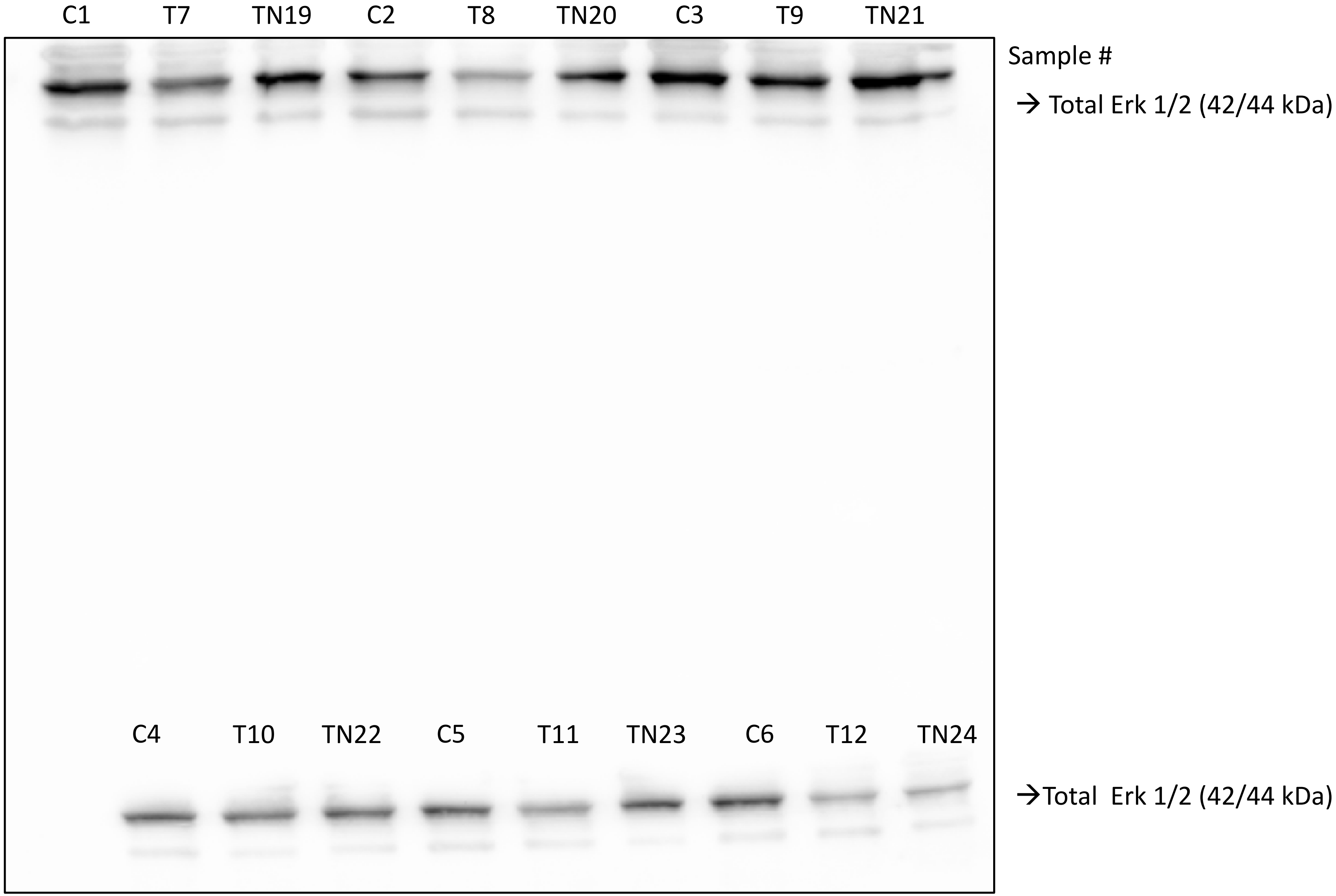

C= unstimulated control
T= stimulated with TGFb 10ng/ml
TN= TGFb + NO_2OA

Conditions:
12,5 µg sample / pocket on 12% Gel
Blot on NC at 200 mA for 2 h
Block 1h in 5% BSA

1st AB:
Total Erk 1/2
1:1000 at 4°C o/n

2nd AB: anti-Rb
In 5% BSA at RT for 2h

Detection: ECL/Femto 4 to 1
Detection time aSMA 10 s

Gene	Forward Primer	Reverse Primer
Actin β	GCTGTATTCCCCTCCATCG	GGGGTGTTGAAGGTCTCAAA
TGFβ	AGCCCGAAGCGGACTACTAT	ACGCCAGGAATTGTTGCTAT
TGFβ R1	TGGTCCAGTCTGCTTCGTCT	GTGGTGCCCTCTGAAATGAA

Supplementary Table S1: Primers used for qPCR
